# A hybrid ensemble learning merging approach for enhancing the super drought computation over Lake Victoria Basin

**DOI:** 10.1038/s41598-024-61520-6

**Published:** 2024-06-15

**Authors:** Priyanko Das, Zhenke Zhang, Suravi Ghosh, Ren Hang

**Affiliations:** 1 Institute of African Studies, School of Geography and Ocean Sciences, Nanjing University, Nanjing, China; 2 Institute of Atmospheric Physics, University of Chinese Academy of Sciences, Beijing, China; 3 Institute of Population Studies, Nanjing University of Post and Telecommunication, Nanjing, China

**Keywords:** Super drought, Ensemble learning, Lake Victoria Basin, Precipitation, Atmospheric science, Climate change, Hydrology, Natural hazards

## Abstract

This study introduces a novel Hybrid Ensemble Machine-Learning (HEML) algorithm to merge long-term satellite-based reanalysis precipitation products (SRPPs), enabling the estimation of super drought events in the Lake Victoria Basin (LVB) during the period of 1984 to 2019. This study considers three widely used Machine learning (ML) models, including RF (Random Forest), GBM (Gradient Boosting Machine), and KNN (k-nearest Neighbors), for the emerging HEML approach. The three SRPPs, including CHIRPS (Climate Hazards Group Infra-Red Precipitation with Station), ERA5-Land, and PERSIANN-CDR (Precipitation Estimation from Remotely Sensed Information using Artificial Neural Network-Climate Data Record), were used to merge for developing new precipitation estimates from HEML model. Additionally, classification and regression models were employed as base learners in developing this algorithm. The newly developed HEML datasets were compared with other ML and SRPP products for super-drought monitoring. The Standardized precipitation evapotranspiration index (SPEI) was used to estimate super drought characteristics, including Drought frequency (DF), Drought Duration (DD), and Drought Intensity (DI) from machine learning and SRPPs products in LVB and compared with RG observation. The results revealed that the HEML algorithm shows excellent performance (CC = 0.93) compared to the single ML merging method and SRPPs against observation. Furthermore, the HEML merging product adeptly captures the spatiotemporal patterns of super drought characteristics during both training (1984–2009) and testing (2010–2019) periods. This research offers crucial insights for near-real-time drought monitoring, water resource management, and informed policy decisions.

## Introduction

Drought, a prevalent natural catastrophe, exerts substantial effects on ecosystems, agriculture, food supplies, industries, and livestock, posing a significant threat to various aspects of life and the environment^1–4^. In the current context, alterations in the occurrence and severity of drought occurrences across diverse regions stem from climate change and escalating water requirements.^5,6^. In the future, regions accustomed to high precipitation levels may unexpectedly experience drought due to climate and land use changes, posing significant challenges for water resource management and environmental protection^7–9^. During the preceding decade (2003–2012), drought events adversely impacted 36.5 million individuals, leading to a substantial loss of life across the globe^10,11^. Additionally, the drought impacts 30% of the world's population and leads to huge economic losses^12^. Hence, precise drought estimation furnishes essential data for policymakers, water managers, and hydrologists to make informed decisions and implement effective water resource strategies^13^. However, drought is classified into four distinct types: agricultural, hydrological, meteorological, and socio-economic. However, drought has been categorized into four types: agriculture, hydrological, meteorological, and socio-economic^14^. The consensus in the scientific community is that all categorised droughts originated from meteorological droughts^15,16^. Over the past few decades, numerous meteorological drought indices have been introduced, such as the Palmer Drought Severity Index (PDSI)^17^ Standardized Precipitation Index (SPI)^18^, and Standardized Precipitation Evapotranspiration Index (SPEI)^19^. Many prior investigations have predominantly focused on the SPI index, which solely captures the impact of precipitation on drought conditions^7,12,20–22^. Conversely, the SPEI incorporates both precipitation and evaporation, crucial factors influencing drought events, providing a comprehensive assessment of surface water balance alterations^14,23^. In addition, the PDSI only reflects the mid-term and long-term drought, whereas the SPEI is suitable for short-term and long-term drought monitoring with better climatic water balance^24–26^. Therefore, this research employs the SPEI for monitoring and predicting meteorological droughts in the Lake Victoria Basin (LVB), East Africa, providing a specialised strategy for regional drought assessment.

Precipitation plays a vital role in the hydrological cycle, forming a connection between the atmosphere, lithosphere, and biosphere, facilitating energy exchange and supporting water resources^6,27–30^. Furthermore, precipitation data serves as the fundamental data source for estimating extreme climate indices, including drought^8^ generally, the occurrence of drought depends on the deficiency of precipitation and the hydrological imbalance of a region^25,31^ Therefore, it is essential to estimate accurate precipitation for drought monitoring and mitigation^32^.Precipitation estimates are predominantly derived from global sources such as satellite-based data, spaceborne weather radar, and ground observations^33,34^. In general, the direct measurements from rain-gauge observation are considered accurate for precipitation estimates but lacking in historical data records, high terrains, and complex topographical regions such as Africa^33,35^. In general, it is important to have one rain gauge (RG) observation for every 575 km^2^, which is challenging in developing countries^36^. In addition, the ground-based observation is sparsely distributed in most developing nations like Africa, and spatial variation of continuous precipitation data based on rain gauge (RG) is subjected to uncertainty^37^. Nevertheless, satellite-based reanalysis precipitation products (SRPPs) offer uninterrupted, long-term regional and global observations with high spatial–temporal resolution^38^. The SRPPs estimate precipitation using passive microwave (PMW) infrared (IR) and space-born radars. The commonly used SRPPs are IMERG (Integrated Multi-Satellite Retrievals for Global Precipitation Measurement)^39^, CHIRPS (Climate Hazards Group InfraRed Precipitation with Station data)^40^, ERA5^41^, TRMM (Tropical Measuring Mission Multi-Satellite Precipitation), TMPA^42^, PERSIANN-CDR (Precipitation Estimation from Remotely Sensed Information using Artificial Neural Network-Climate Data Record)^43,44^. However, it should be noted that the sampling error, different retrieved algorithms, and various sensors are the major cause of biases in SRPPs^41^. Hence, various algorithms have been created to amalgamate these SRPPs, aiming to enhance the precision of precipitation estimates^11,37^.

Over the recent decades, Machine Learning (ML) algorithms have been globally employed to merge various SRPPs, enhancing the accuracy of precipitation estimates. Zhang et al.^37^ introduced innovative dual Machine Learning (ML) algorithms utilising Random Forest (RF), support vector machine (SVM), extreme learning machine (ELM), and artificial neural network (ANN) to merge four satellite precipitation products across China. Lin et al.^45^ applied three Machine Learning (ML) models—Random Forest (RF), Decision Tree (DT), and Adaptive Boosting Decision Trees (Adaboost)—to rectify and analyse errors in satellite precipitation estimates within the Three Gorges Reservoir Area (TGRA). Zandi et al.^46^ proposed a stacked generalisation ensemble approach consisting of RF, SVM, and MLP (Multilayer perceptron) neural network for developing new high-resolution precipitation estimates and compared to LWLR (local weighted linear regression) approach in a topographically complex region. In a recent investigation conducted by Ghosh et al.^11^, dual Machine Learning (ML) algorithms, including Gradient Boosting Machine (GBM), Random Forest (RF), Support Vector Machine (SVM), and k-nearest Neighbors (KNN), were employed for the purpose of monitoring drought conditions in Kenya. However, most of the previous studies only focused on developing new precipitation estimates using different ML models and did not justify the capacity of these precipitation data for extreme climate event detection, especially drought in East Africa.

This study addresses a critical gap in existing research by focusing on the application of new precipitation estimates for extreme climate event detection, particularly drought, with a specific emphasis on super drought events. Although previous studies primarily focused on developing precipitation estimates using machine learning (ML) models without evaluating their suitability for extreme climate event detection, our research takes a pioneering approach. This study introduces a hybrid ensemble ML merging approach that amalgamates Satellite-based Reanalysis Precipitation Products (SRPPs) with consideration of other topographical and climatic factors. By integrating these additional factors into the ML merging process, this study aims to enhance the accuracy and reliability of the precipitation estimates, particularly in capturing extreme climate events such as super droughts. The primary objective of this study is to develop and evaluate new precipitation estimates derived from the hybrid ML merging approach. This study assesses the capacity of these estimates for super drought estimation, including aspects such as frequency, intensity, and duration, over the Lake Victoria Basin (LVB). This comprehensive evaluation fills a significant gap in the literature, providing valuable insights into the characteristics and performance of the new precipitation estimates in capturing extreme climate events in the Lake Victoria Basin.

Over the recent decades, the Lake Victoria Basin (LVB) has encountered severe climatic incidents, notably a catastrophic drought in the early 1980s, significantly impacting the flow of the Nile River at the basin's outlet^47^. The drought events in LVB have been associated with water scarcity, ecosystem degradation, and crop failure, indicating the urgent need for a reliable drought prediction system. In addition, the LVB faces various challenges to the disappearance of water bodies under climate change conditions and anthropogenic forcing^48^. This dry condition affected 30 million people and agriculture production in LVB^49–51^. Although existing methods for estimating precipitation from Rain Gauge (RG) observation and SRPPs often suffer from accuracy and spatial coverage limitations, especially in complex terrain and sparse regions. Despite the challenges posed by low RG density and incomplete historical records, Das et al.^7^ assessed two long-term satellite precipitation products in the Lake Victoria Basin (LVB), revealing substantial uncertainties in the estimation of drought characteristics. In response to these challenges, this study proposed the development of a hybrid ensemble ML merging algorithm, which represents a significant advancement in drought monitoring. However, no studies were found to estimate super drought characteristics in LVB. This is the first study that tested ensemble ML model capacity for capturing the super drought frequency, intensity, and duration.

This study proposed a Hybrid Ensemble ML (HEML) algorithm for merging three SRPPs and developed new precipitation estimates based on three widely used ML algorithms, including RF, KNN, and GBM. Utilizing this updated precipitation estimate, the analysis involved the computation of super-drought events and their corresponding characteristics within the Lake Victoria Basin (LVB). Six statistical metrics were employed in the assessment process to validate the HEML product. The primary aim of this study is (i) To appraise the HEML precipitation estimates alongside three SRPPs products, CHIRPS, PERSIANN-CDR, and ERA5-Land, and to compare them with RG observations and (ii) To assess the efficiency and dependability of HEML products in computing super droughts and to compare them with SRPPs products. (iii) To assess the precision of the HEML product in capturing the frequency, intensity, and duration of super droughts in the LVB. This new hybrid algorithm would be used to develop new precipitation estimates for drought monitoring at the basin scale. These research findings are relevant and applicable to various stakeholders involved in water resource management, climate monitoring, and disaster preparedness within the Lake Victoria Basin (LVB) region. Specifically, governmental agencies, policymakers, hydrologists, climatologists, and researchers working on drought monitoring and prediction will find our results valuable for informing decision-making processes, implementing mitigation strategies, and enhancing resilience to extreme climate events, particularly super droughts.

## Experimental design and methodology

### Victoria Lake Basin

**Figure 1:**
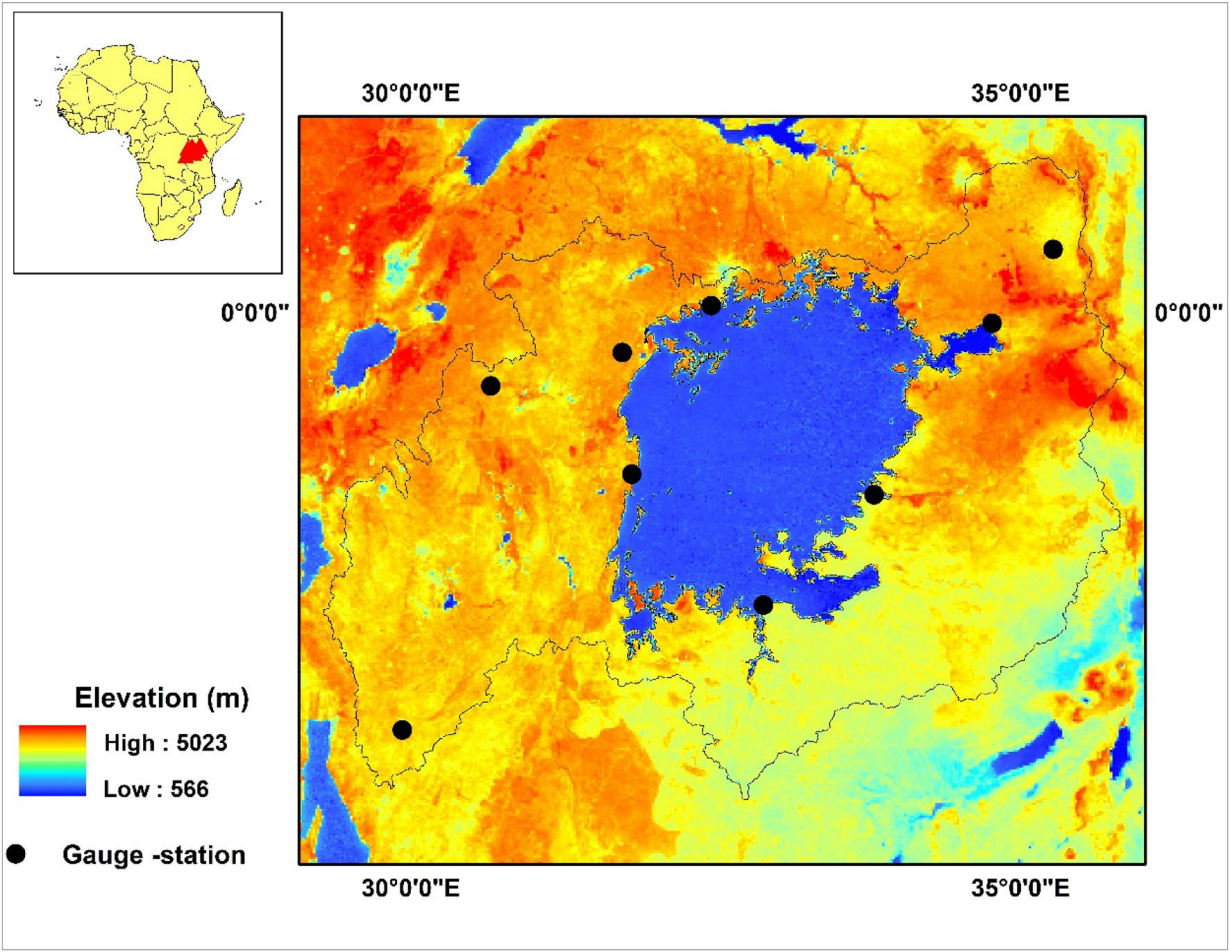
Location map of the Lake Victoria Basin with Gauge station.

Lake Victoria, the largest freshwater lake in Africa, plays a crucial role in providing ecosystem services to five African nations: Kenya, Rwanda, Uganda, Tanzania, and Burundi, as illustrated in Fig. 1^52^. The Lake Victoria Basin (LVB) spans 194,000 square kilometres in the western part of the Rift Valley^7^. The LVB is influenced by climate change and is considered a severe risk region due to anthropogenic force^48^. The precipitation pattern in LVB follows a bi-modal distribution, consisting of a long rainy season (March, April, and May—MAM), a short rainy season (September, October, and November—SON), and a dry season (June, July, August—JJA)^49,53,54^. The annual average rainfall in the region ranges from 880 to 2600 mm, with temperatures varying between 15 ℃ in high-altitude areas to 28 ℃ in semi-arid regions^47^. Furthermore, the inter-annual and seasonal variability is shaped by the inter-tropical convergence zone (ITCZ) and El Niño/Southern Oscillation (ENSO), as documented by Kizza et al.^55^. Table 1 presents the climatic and topographical features of the rain gauge (RG) network across the Lake Victoria Basin (LVB).

**Table 1:** Description of climatic features and geography of Rain—gauge station.

Station Name	Latitude	Longitude	Elevation (m)	Average temperature (℃)	Annual precipitation (mm)
Bukoba	− 1.33	31.82	1160	22.41	2145
Eldoret	0.514	35.27	6857	17.5	1103
Entebbe	0.052	32.46	3871	22.2	1640
Gitega	− 3.429	29.93	4934	25	1305.2
Kisumu	− 0.0917	34.76	3711	24.6	1966
Masaka	− 0.33	31.73	1288	22.3	1383
Mbarara	0.607	30.65	3763	22.01	1029
Musoma	− 1.5	33.8	3720	23.6	1317
Mwanza	− 2.4	32.89	1261	23.2	1050

### Datasets

This study considers three SRPP products from 1984 to 2019, including CHIRPSv2, PERSIANN-CDR, and ERA5-Land (ERA-5L), for drought monitoring due to their spatial and temporal resolution, which is essential for capturing precipitation patterns. It should be noted that drought estimation requires long-term datasets (> 30 years) on a monthly scale, especially for SPI/SPEI computation^18,56^.These three long-term SRPPs provide valuable opportunities to evaluate drought from a climatological perspective, especially for sparse gauge networks, such as East Africa^57^. In addition, previous studies (Table S1) extensively consider these SRPPs for drought monitoring in similar climate characteristics.

#### Rain-gauge-based gridded data

This study considers CRU-TS (Climate Research Unit-Time Series) version 4 datasets based on RG observation to evaluate SRPPs and HEML products in LVB. The CRU-TS provides monthly gauge data with no high-resolution missing records, which is important for drought computation. The datasets used the ADW (angular distance weighting) interpolation method to gridded 0.5°grid with excellent quality control and homogeneity check and were available from 1901 to 2020^58^. This maintains good data quality for drought computation. Previous studies extensively used CRU-TS datasets for evaluating the satellite precipitation products and global climate models for extreme event monitoring^7,11,25,59,60^. Thus, this study uses CRU-TS data to evaluate the SRPPs and HEML precipitation estimates for drought monitoring. The CRU-TS data was retrieved from the University of East Anglia website (https://www.uea.ac.uk/groups-and-centres/climatic-research-unit) from 1984 to 2019.

#### CHIRPS

The CHIRPS dataset, utilised for drought monitoring and prediction, is produced collaboratively by the Climate Hazards Group (CHG), the University of California, Santa Barbara (UCSB), and the U.S. Geological Survey (USGS)^8,40,61^. The datasets were integrated with Cold Cloud Duration (CCD) derived from gauge observations and satellite estimates, providing quasi-global coverage from 50° N to 50° S, and are accessible from 1981 to the present^40,62,63^. This study selects newly developed CHIRPS version-2 precipitation estimates available on a monthly scale with high spatiotemporal resolution (0.05° × 0.05°)^64^. Therefore, this study considers CHIRPS datasets when merging algorithms for super-drought monitoring. The datasets are freely available on the CHG website (https://www.chc.ucsb.edu/data/chirps).

#### PERSIANN-CDR

The precipitation estimates from PERSIANN-CDR were formulated under the National Climate Data Centre (CDC) and Climate Data Record (CDR) program at NOAA (National Oceanic and Atmospheric Administration) in collaboration with the Center for Hydrometeorology and Remote Sensing (CHRS) at the University of California^43,57,65^. The datasets provide long-term precipitation estimates with high spatial resolution (0.25° × 0.25°) and cover quasi-global coverage of 60° N × 60° S and available from 1983 to 2019. Furthermore, this dataset is derived from Gridsat-B1 IR data and corrected using precipitation estimates from the Global Precipitation Climatology Project (GPCP)^66^. The datasets are freely available on the CHRS website (http://chrsdata.eng.uci.edu/).

#### ERA5

The ERA5-Land reanalysis dataset is produced through an Integrated Forecasting System (IFS) using a 4D-var assimilation algorithm in version 41r2 by the European Centre for Medium-Range Weather Forecast (ECMWF)^67^. The datasets have been available from 1950 to the present with high spatiotemporal resolution (0.1° × 0.1°) at a global scale^11^. The ERA5-L products contain different climatic parameters, including precipitation estimates. Thus, this study considers ERA5-L precipitation datasets with other influencing climatic factors^4^ max and min temperature, surface pressure, latent heat, specific humidity, and longwave radiation) on the HEML algorithm. The ERA5-L products are downloaded from the ECMWF website (https://confluence.ecmwf.int/site/support). The satellite precipitation products utilized in this study are detailed in Table 2.

**Table. 2.** Detail description of satellite-based reanalysis precipitation products (SRPPs).

Satellite products	Study period	Spatial resolution	Spatial coverage
CHIRPSv2	1983–2019	(0.05° × 0.05°)	quasi-global coverage from 50° N to 50° S
PERSIANN-CDR	1981–2023	(0.25° × 0.25°)	cover quasi-global coverage of 60° N × 60° S
ERA5-Land	1950–2023	(0.1° × 0.1°)	Global scale

### Methods

#### SPEI

In this research, the calculation of drought involves the utilisation of the Standardized Precipitation Evapotranspiration Index (SPEI). This index provides insights into the study region's water deficit and overall water balance^4,23,68^. This formula integrates the Standardized Precipitation Index (SPI) with the Palmer Drought Severity Index (PDSI) sensitivity to variations in evaporation caused by temperature changes^19,68^. At first, the monthly potential evapotranspiration (PET) is computed, and the difference between the monthly water balance of each month (i.e., deficit ${\omega }_{i}$) for estimating SPEI and then subtracting PET value from precipitation of the given month (ρ_i_)^14^ This study used the Thornthwaite algorithm for the computation of PET^69^. The SPEI is expressed as:

1 $${\omega }_{i} = {\rho }_{i}-{PET}_{i}$$

Here, ρ represents precipitation, and PET stands for potential evapotranspiration.

2 $$f\left(\gamma \right)={\left[1+ {\left(\frac{\alpha }{\gamma -\beta }\right)}^{\varepsilon }\right]}^{-1}$$

3 $$SPEI=W- \frac{{X}_{0}+{XW}^{1}+{XW}^{2}}{1+{Z}_{1}W+{Z}_{2}{W}^{2}+{Z}_{3}{W}^{3}}$$

4 $$W= \sqrt{-2{\text{ln}}(p)}$$

where $\beta$, $\alpha$, and $\varepsilon$ represent the shape, scale, and origin. The SPEI scale refers to two conditions: (i) p ≤ 0.5, p = 1 − $f\left(\gamma \right)$ and (ii) p > 0.5, p = 1 − p. The ${X}_{0} , {X}^{1}, and {X}^{2}$ are constant values which equal to 2.51551, 0.80285 and 0.01032. Similarly, the constant values of ${Z}_{1}, {Z}_{2}, and {Z}_{3}$ are 1.43278, 0.18926, and 0.00130, respectively. This study incorporates three SPEI time scales: SPEI-3, SPEI-6, and SPEI-12, representing short-term, medium-term, and long-term drought, respectively.

#### Super drought events and their characteristics

The term “super drought” denotes the incidence of severe or extreme drought events across multiple time scales^70,71^. Typically, the impact of a multi-scale drought event is reflected in the response of available water resources, such as river discharge, soil moisture, and reservoir storage^56^; this complexity poses a challenge in accurately defining extreme drought events within a region^72^. Therefore, Wang et al.^73^ developed the Super drought concept, which is effective for showing the severe/extreme drought condition at a regional scale^72^. A details explanation of the super drought concept and method has been found in Wang et al.^72,73^. The super drought is characterized by SPEI values consistently below − 1.5 across multiple timescales, including SPEI-3, SPEI-6, and SPEI-12^70,71^. However, the super drought has been classified into two categories: (i) SE type (all the SPEI values are lower than − 1.5) and (ii) E type (all the SPEI values are below − 2). In general, the occurrence of E-type drought is 0.1%, and sufficient samples of RG stations are needed. Thus, this study only considers SE type for super drought computation in LVB.

**Figure 2:**
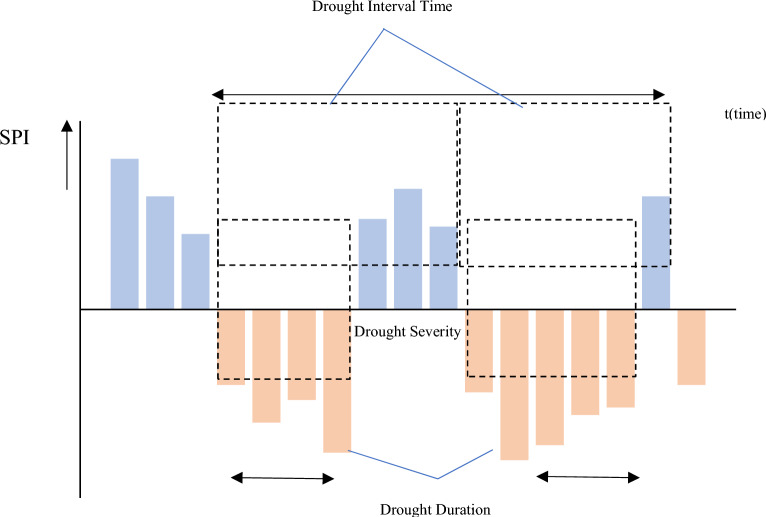
Theory of run model used in this study.

This study used run theory to estimate the super drought frequency, duration, and intensity based on three SPEI time scales^74^. The theory of the run model selects a threshold value that determines the start of a drought or dry period^75^. The drought period re-starts when the SPEI index values are below the threshold value and continue to be greater than the threshold value shown in Fig. 2.

The frequency of drought occurrence is defined as:

$${f}_{g }= \frac{{\rho }_{g }}{{\tau }_{g}} \times 100\mathrm{\%}$$

where $f$ represents the frequency, ${\rho }_{g}$ represent the number of super drought events (< − 1.5), ${\tau }_{g}$ represent the sum of all the months during the study period. g represents the grid cell.

The drought duration is defined as:

$$DD = \frac{\sum_{i=1}^{n}{d}_{l}}{\epsilon }$$

where DD represent the drought duration, ${d}_{l}$ represent the duration of *l*th drought event, and $\epsilon$ represent the sum of all drought months.

The drought Intensity is defined as:

$$DI = \left|\frac{1}{n} \sum_{i=1}^{n}{SPI}_{l} \right|$$

where DI represents the drought intensity, n represents the number of super drought events that occur in months with SPEI < − 1.5, and ${SPEI}_{l}$ represent the less-than-threshold value.

#### Hybrid Ensemble machine learning (HEML) approach for merging SRPPs

Ensemble machine learning was created by combining various machine learning (ML) algorithms to enhance predictive performance^4,76^. In general, the ensemble ML model uses a meta-learner and base learner to train the individual train algorithm and then perform validation for prediction. The data has been split into k-folds to avoid overfitting input data, and K-1 folds are used for training the base learners^46^.This study considers three widely used ML models, including RF, KNN, and GBM, and six drought-influencing climatic and topographical factors including PET and temperature. for developing the HEML model for merging three SRPPs datasets. These three algorithms have been extensively employed to enhance precipitation estimates in various research studies^11,41,66,77,78^. The Random Forest (RF) algorithm, utilising the decision tree method, has been identified as the optimal ML algorithm for merging based on its ability to enhance generalization capacity, mitigate overfitting issues, and employ ensemble predictors through random selection^9,79^. The K-Nearest Neighbors (KNN) approach identifies “K” training points closest to the hypothetical query point (Y) and classifies them based on the majority, making it a prevalent method for classification^80^. The Gradient Boosting Machine (GBM) algorithm is employed to minimise the disparity between predicted and observed values through a tree-based ensemble learning approach, as outlined by Friedman in^81^. Thus, this study stacked all these ML algorithms to develop a new merging approach.

**Figure 3:**
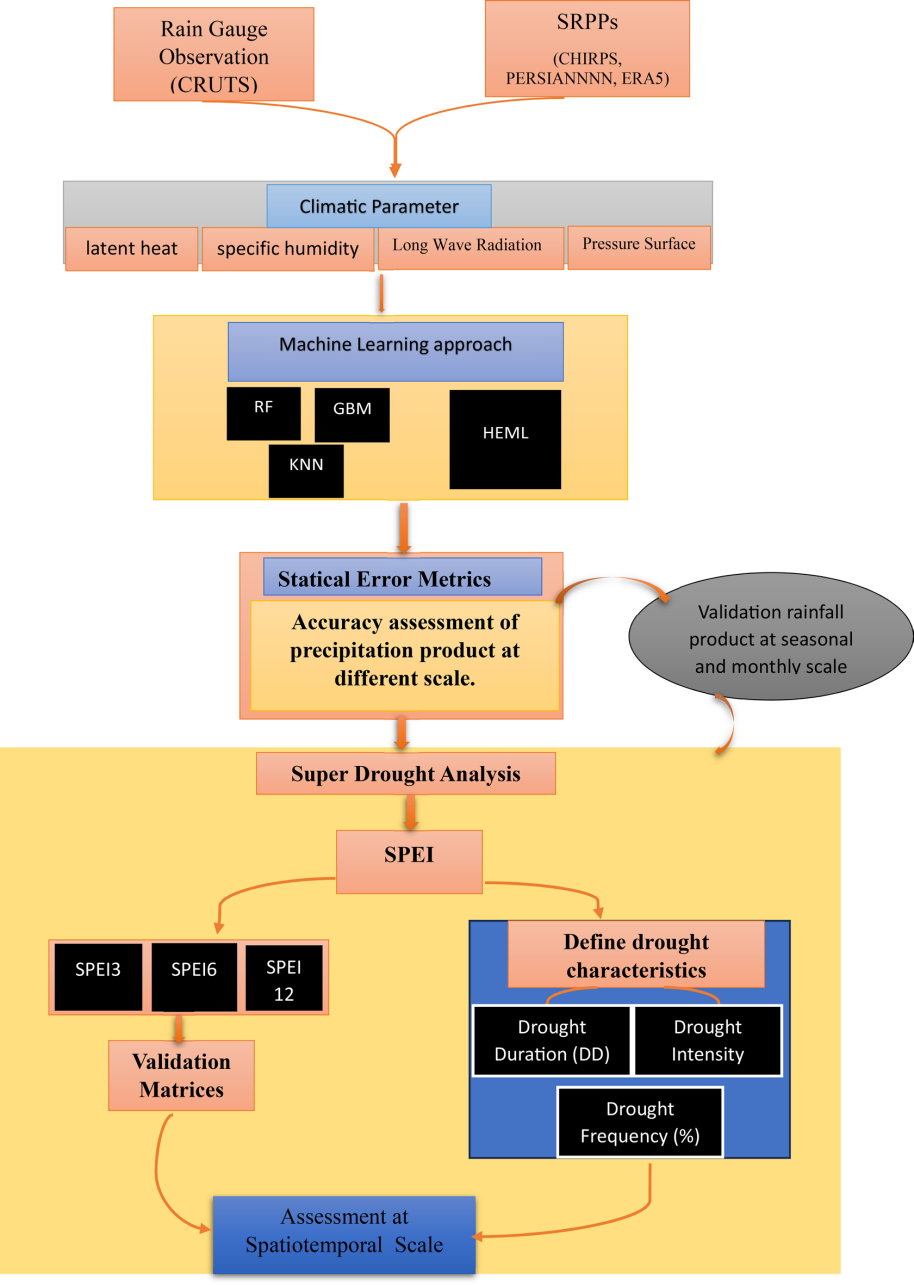
Flowchart of methodology.

Furthermore, the utilisation of the classification and regression model in base learners was chosen for its superior performance in comparison to the sole reliance on the regression model^11,66^.The implemented HEML model workflow is illustrated in Fig. 3. The precipitation datasets have been divided into 70% for training and 30% for testing from 1984 to 2019. The hyperparameters of the HEML algorithm, including ntree, interaction depth, shrinkage, and number of folds, are detailed in Table 3.

**Table 3:** Details d of ML and HEML models used in this study.

ML model	Parameters
Hybrid ensemble machine learning (HEML)	HEML = (RF + GBM + KNN)
Random forest (RF)	Number of trees (ntrees) = 300, Leaf Node ($${n}_{min})$$, number of features $$\left({n}_{feature}\right)=2$$,
K-nearest neighbour (KNN)	Number of k = 1:16 Method = “repeatedcv)” K fold = 6
Gradient boosting machine (GBM)	Ntree (α) = 600, I nteraction. depth = 3, Shrinkage = 0.001, n. minobsinnode = 0.5

#### Statistical evaluation

This study used six statistical metrics to validate the newly developed HEML model results and the capability of capturing the super drought characteristics and comparing them with other SRPPs. These include Correlation coefficient (CC), modified Kling-Gupta Efficiency (MKGE), Theil's U, Heidke skill score (HSS), Root Mean Square Error (RMSE), Relative Absolute Error (RAE), and Mean Absolute Error (MAE). The CC was used to establish the relationship between predicted and observed values and quantify the model's strength. The MKGE score described the relationship between observed and predicted time series based on bias ratio (β), Variability ratio (γ), and liner correlation (r)^82^. Theil's U score evaluates the performance of predicted values, especially for precipitation estimates^83^.The HSS indicates the relative enhancement of the forecast compared to the standard prediction. The RMSE, RAE, and MAE scores show the magnitude and systematic error between predicted and observed values. The specific algorithm employed for estimating the statistical metrics is outlined in Table 4.

**Table. 4.** Details of validation metrics used for accuracy assessment.

Metrics	Equation
MKGE	$1-\sqrt{{\left(cor-1\right)}^{2 }+\left(\frac{\propto \left(E\right)}{\propto \left(o\right)}\right){-1}^{2}+{\frac{\beta (E)/\propto (E)}{\beta (O)/\propto (O)}-1)}^{2}}$
Theil’s U	$\sqrt{\frac{1}{n}} {\sum }_{i=1}^{n}{(E}_{i}-{O}_{i}{)}^{2}/{\sum }_{i=1}^{n}{E}_{i}^{2}$
MAE	$\frac{1}{n}\sum \langle {E}_{i}-{O}_{i}\rangle$
RAE	$\frac{\sum ({E}_{i-}{{O}_{I})}^{2}}{\sum {O}_{i}^{2}}\times 100$
COR	$\frac{{\sum }_{i=1}^{n}\left({O}_{i}-\overline{O }\right)\left({E}_{i}-\overline{E }\right)}{\sqrt{\sum_{i=1}^{n}\left({O}_{i}-\overline{O }\right)}\sqrt{\sum_{i=1}^{n}\left({E}_{i}-\overline{E }\right)}}$
HSS	$\frac{2(PS-QR)}{\left(P+R\right)\left(R+S\right)+(P+Q)(Q+S)}$

“O” observation, “E” prediction, “n” number, “P” hits, “Q” false alarms, “R” misses, “S” correct negatives.

In this study, data analysis and visualization were conducted using R version 4.3, employing several key packages including ggplot2, MLmetrics, hydroGOF, metaEsenmble, and DescTools. Additionally, ArcGIS version 10.8 was utilized for map layout.

#### Ethical approval

All the work complies with Ethical standards.

### Results

#### Valuation of precipitation estimates

**Figure 4:**
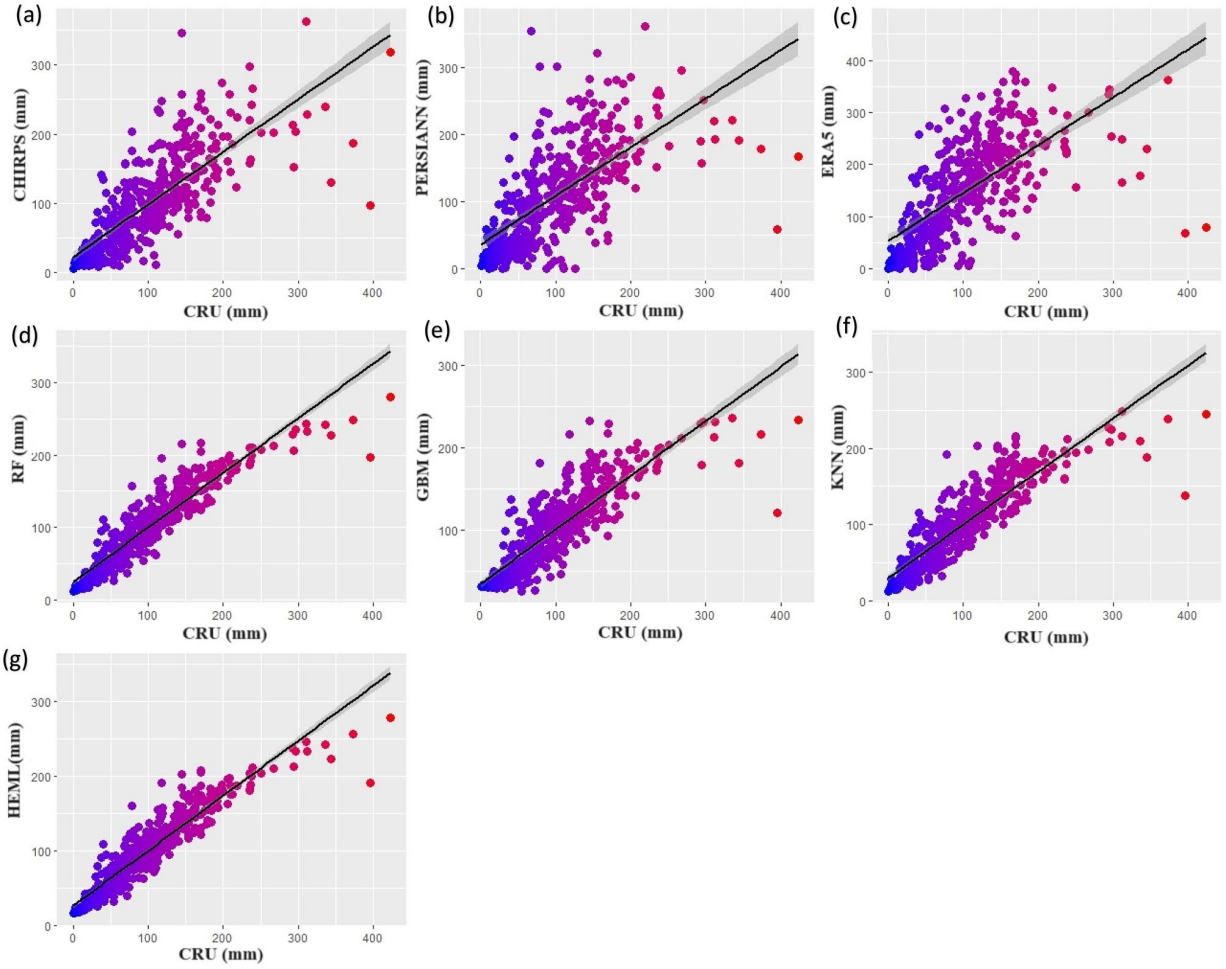
Scatter diagram showing the relationship between ML, SRPP products and newly developed HEML products with observation datasets.

The density scatter plot shows the linear relationship between newly developed HEML precipitation estimates and observation data and compares them with other SRPPs and ML products in LVB (Fig. 4a–g). The results indicate that the SRPP values were overestimated/underestimated compared to observation. In contrast, the ML and HEML results show less overestimated/underestimated precipitation estimates. Generally, seven statistical metrics were used to define precipitation products' relationship and uncertainty, including MKGE, Theil'U, CC, RMSE, RAE, HSS, and MAE, during the testing period (2010–2019). Among the SRPPs datasets, CHIRPS products show the best performance (CC = 0.77, HSS = 0.63), followed by ERA5 (CC 0.66, HSS = 0.57) and PERSIANN-CDR (CC = 0.65, HSS = 0.56) (Fig. 4a–c) (Table 5). although, the ERA-5 shows large biases (RMSE = 82.79, MAE = 62.3) in precipitation estimates then CHIRPS (RMSE = 44.21/mm, MAE = 29.9) and PERSIANN-CDR (RMSE = 58.88/mm, MAE = 40.8). The single merging algorithm improved the precipitation estimates and reduced the uncertainty between observed and estimated products. The RF algorithm shows better performance (CC = 0.92, HSS = 0.81, RMSE = 0.26/mm) compared to SRPPs datasets followed by KNN (CC = 0.88, HSS = 0.76, RMSE = 32.49/mm) and GBM (CC = 0.85, HSS = 0.71, RMSE = 35.49/mm) (Fig. 4d–f). However, the new HEML precipitation product shows excellent performance (CC = 0.93, HSS = 0.84) and reduces the biases of precipitation estimates (RMSE = 26/mm, MAE = 17.4) (Fig. 4g). The MKGE score shows that the HEML algorithm improved the precipitation estimation from 0.43 to 0.8 and reduced the error from 62/mm to 26/mm (Fig. 4g).

**Table 5:** Details description of error metrics.

Evaluation metrics	CHIRPS	PERSIANN	ERA5	RF	HEML	KNN	GBM
CC	0.77	0.65	0.661	0.92	0.93	0.88	0.85
RMSE	44.21	58.88	82.79	26.58	26.05	32.23	35.49
MAE	29.99	40.82	62.30	17.44	17.32	21.26	24.08
MKGE	0.77	0.64	0.44	0.80	0.78	0.76	0.73
THEILU	0.36	0.49	0.68	0.22	0.22	0.26	0.29
RAE	0.59	0.80	1.2	0.34	0.33	0.41	0.47
HSS	0.63	0.56	0.57	0.81	0.84	0.76	0.71

**Figure 5:**
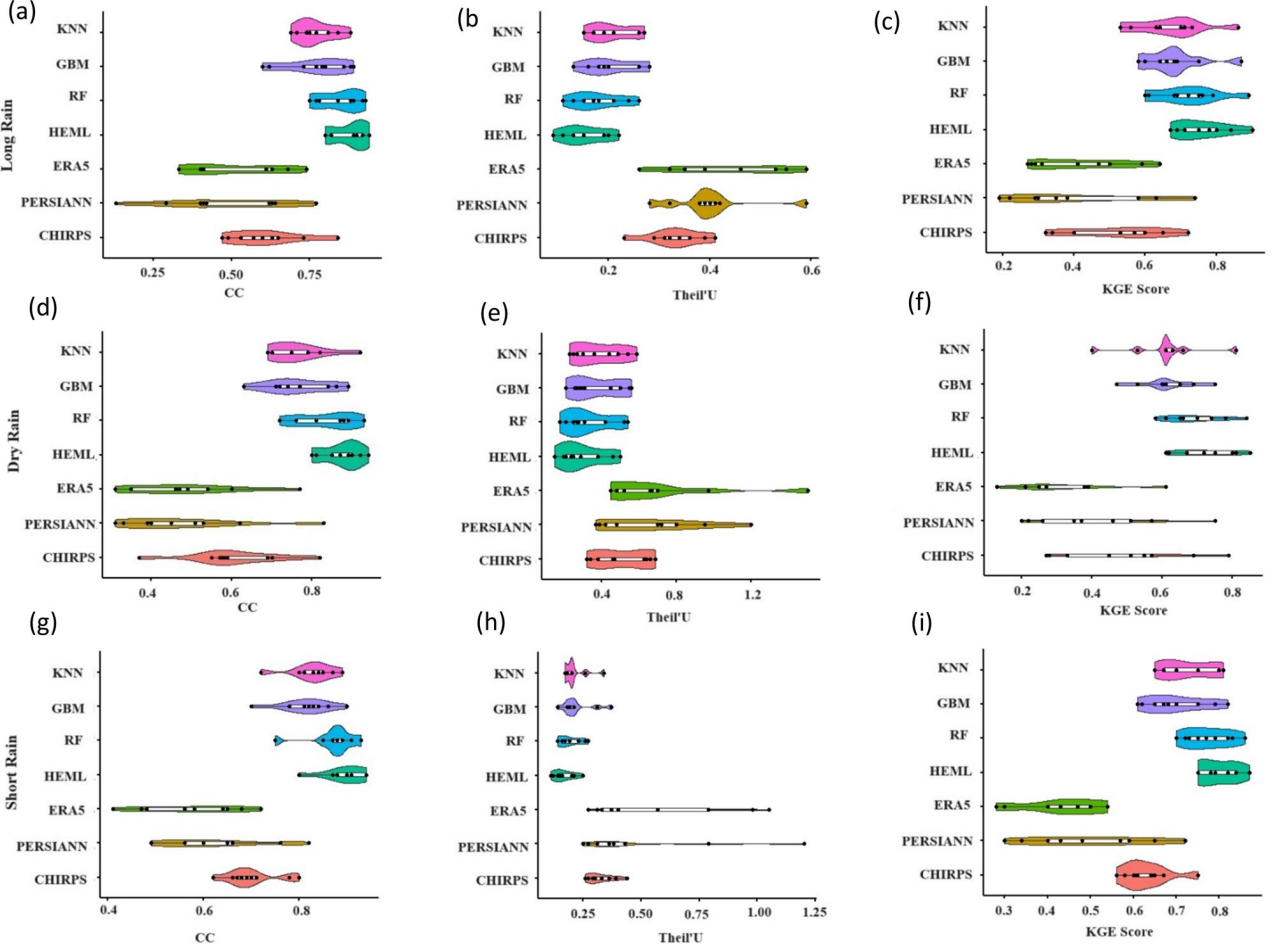
Violin charts of statical error metrics including CC, Theil' U, MKGE Score of Satellite Precipitation Products and single Machine learning product and HEML products. (**a**) Long Rain (CC), (**b**) Long Rain (Theil's U), (**c**) Long Rain (MKGE Score), (**d**) dry rain CC, (**e**) Dry Rain (Theil's U), (**f**) Dry Rain (MKGE Score), (**g**) Short rain (CC), (**h**) Short Rain Theil's U, (**i**) Short Rain (MKGE Score).

The violin plot in Fig. 5 illustrates the progression of SRPPs and ML precipitation estimates during one dry season and two rainy seasons (MAM and short OND). This is assessed through three continuous statistical metrics, CC, MKGE, and Theil’s, across the Lake Victoria Basin during the testing period (2010–2019). The SRPPs perform better in two rainy seasons than in the dry season. The CHIRPS products demonstrate a favourable correlation with observational data for the long rain (CC = 0.6, MKGE = 0.52), short rain (CC = 0.7, MKGE = 0.63), and dry season (CC = 0.6, MKGE = 0.49) in contrast to PERSIANN-CDR and ERA5-L (Fig. 5a–i). At the same time, the ERA5-L products show very weak performance for capturing the dry season (CC = 0.47, MKGE = 0.3, Theil'U = 0.7) in LVB. However, the ML merging algorithms have improved the precipitation estimate for all the seasons in LVB. The CC of the ML merging approach is higher than SPRPPs in the long rain (RF = 0.84, KNN = 0.77, GBM = 0.77), short rain (RF = 0.87, KNN = 0.82, GBM = 0.81), and dry season (RF = 0.83, KNN = 0.76, GBM = 0.76) (Fig. 5a,d,g). Similarly, the MKGE score has improved after fusing SPRPPs using ML algorithms for all seasons. The newly developed HEML merging approach shows excellent performance compared to SPRPPs and single ML precipitation estimates in all the seasons over LVB (Fig. 5a–i). The CC score has been improved for long rain (HEML = 0.88), short rain (HEML = 0.9), and dry season (HEML = 0.85). It should be notable that the SPRPPs, KNN, and GBM merging approach shows poor performance in the dry season, while RF (MKGE = 0.69, Theil'U = 0.33) and HEML (MKGE = 0.72, Theil'U = 0.29) improved the precipitation estimates in the dry season and closed to observation.

### Evaluation of SPEI

**Figure 6:**
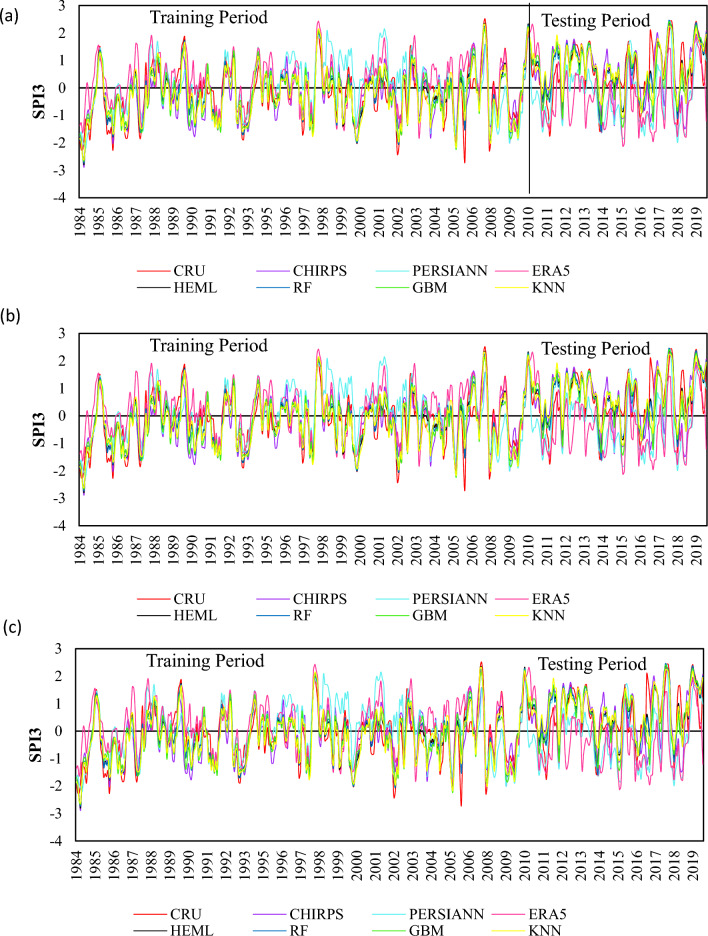
Variation of SPI3, SPI6 and SPI12 timescale based on satellite products and HEML and ML products (1984–2019).

The SPEI was estimated from SRPPs, single ML, and HEML precipitation estimates at a three-time scale (SPEI-3, SPEI-6, SPEI-12) during the training (1984–2009) and testing period (2010–2019) and compared to observed SPEI values over LVB. These three-time scales represent the three types of meteorological drought condition, which includes short (SPEI-3), medium (SPEI-6), and long-term (SPEI-12) draught and affected the water resource of the region (Das et al., 2022). Figure 6 indicates the performance of SRPPs, ML, and HEML estimated SPEI values during the training and testing period at three time scales. In general, the HEML estimated SPEI values show excellent performance and are very close to observation for all the time scales. Among the SRPPs, estimated SPEI shows the worst performance for capturing drought events with overestimated/underestimated (Fig. 6a–c). For example, the drought event that started in 1984 was overestimated by SRPPs for SPEI-12 (Fig. 6c). The SRPPs estimated SPEI values fluctuate more and indicate overestimation/underestimation, especially for short-term drought (SPEI-3). Figure 6c demonstrates that the ERA-5 and PERSIANN-CDR are overestimated/underestimated for capturing the SPEI-12. While the ML merged estimated SPEI values are less fluctuated and closed with observation at all the SPEI timescale. Although the HEML estimated SPEI value achieved excellent accuracy and closed with observation values, it shows the capturing ability of drought events in LVB.

**Figure 7:**
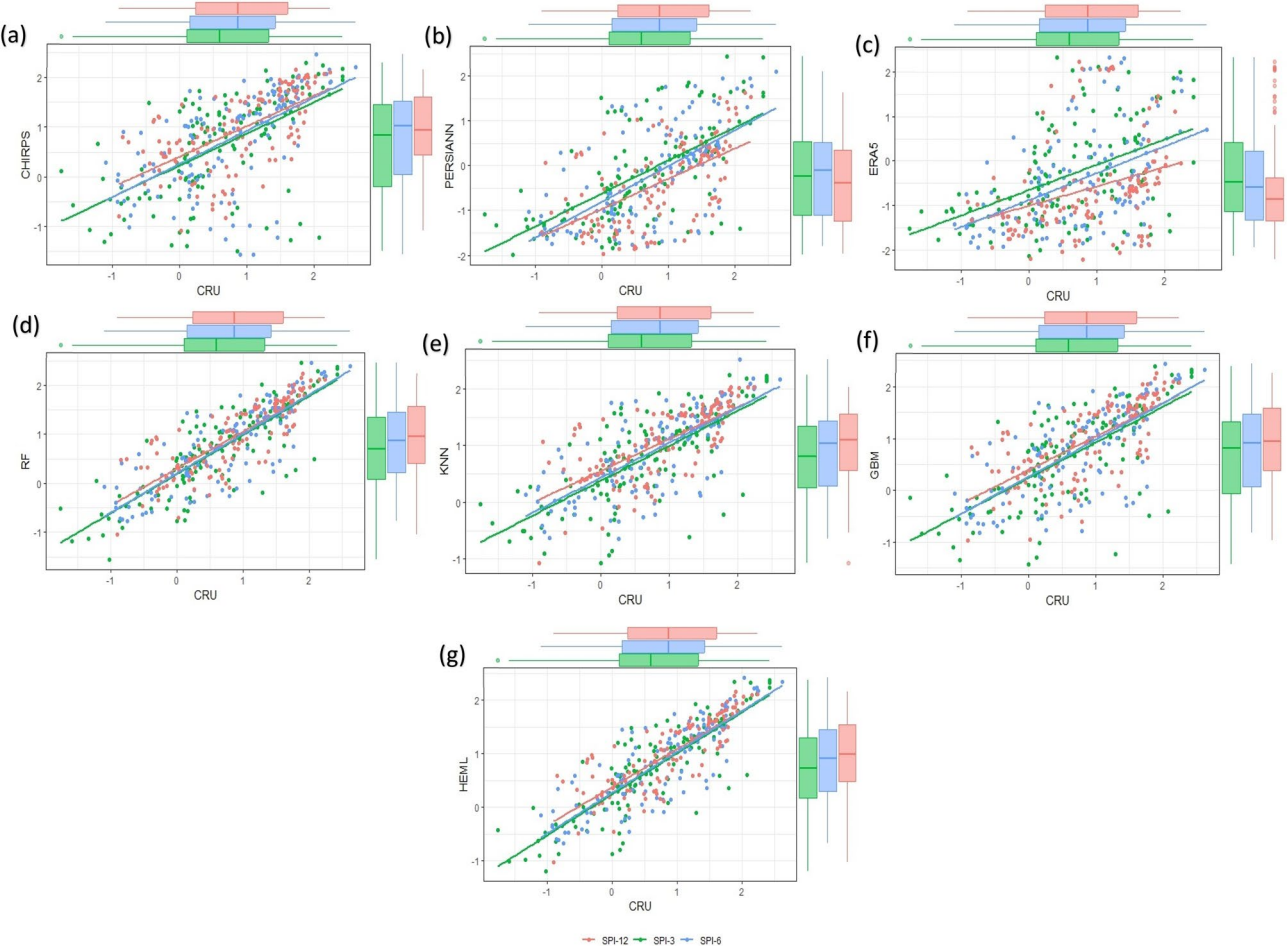
Scatter plot illustrate the correlation between predicted and observed SPEI at multi-scale (SPEI-3, SPEI-6, SPEI-12) during the testing period (2010–2019).

**Figure 8:**
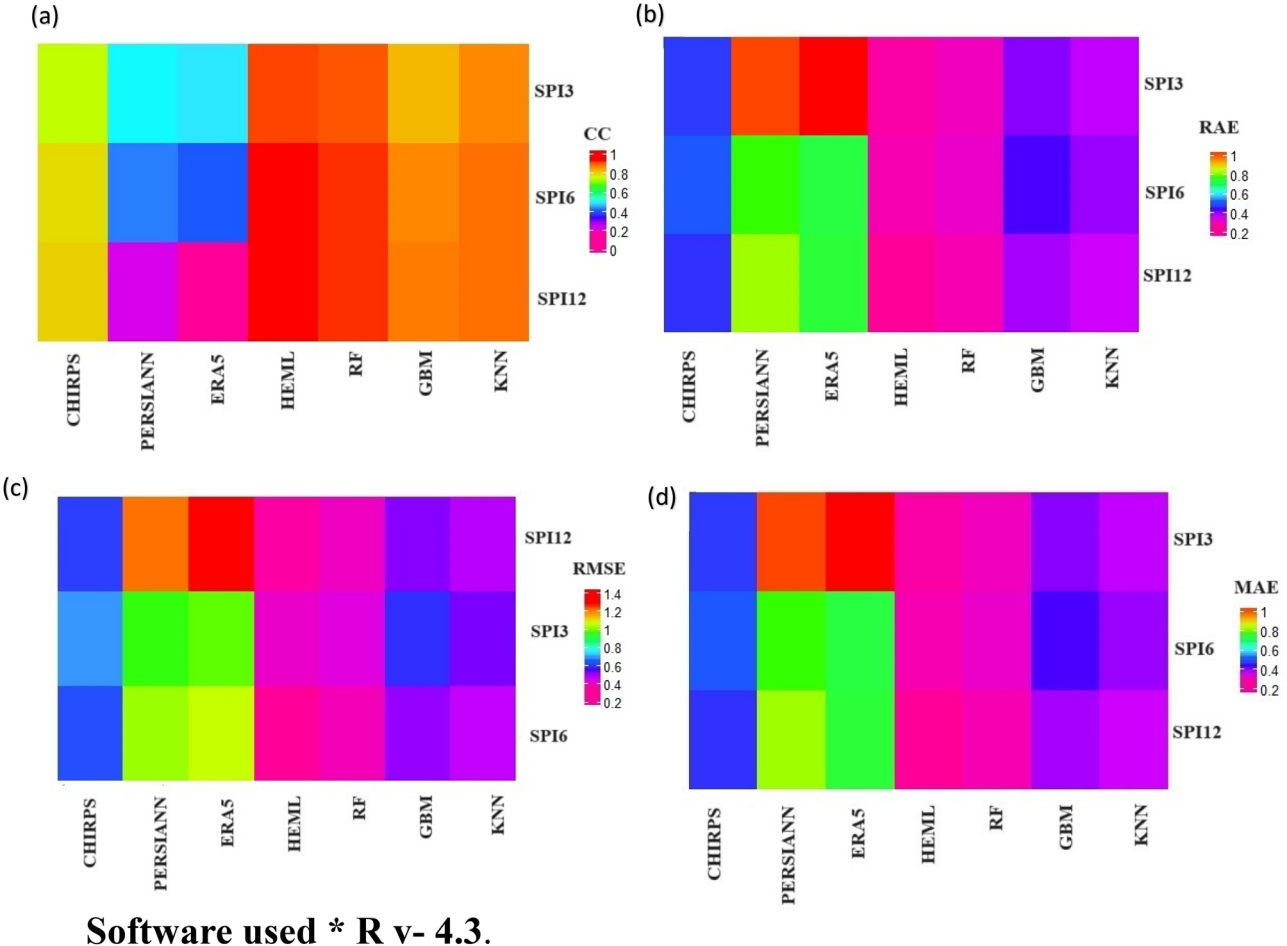
Heat map of (**a**) CC (**b**) RAE (**c**) RMSE (**d**) MAE indicating the accuracy between HEML and satellite and Machine Learning Products using R v-4.3.

Figure 7 illustrates the scatter plot depicting the relationship between predicted and observed SPEI at multiple scales (SPEI-3, SPEI-6, SPEI-12) throughout the testing period. The results indicate that the SRPPs estimated SPEI values are overestimated/underestimated compared to observation at all the time scales. Among the SRPPs, ERA5-L shows a poor linear relationship and cannot be used for drought monitoring in LVB (Fig. 7c). In addition, the RF, GBM, and KNN improved the SPEI values and best linear relationship with observation (Fig. 7d–f). However, the HEML estimated SPEI values show an excellent linear relationship compared to SRPPs and other ML merging products (Fig. 7g). Furthermore, Fig. 8 presents four error metrics, CC, MAE, RAE, and RMSE, evaluating the SPEI estimates at various time scales from SRPPs, single ML, and HEML. CHIRPS' SPEI (3,6,12) exhibits a robust relationship (CC = 0.78) and lower error (RMSE = 0.63, MAE = 0.47) associated with PERSIANN-CDR and ERA5-L (Fig. 8c,d). The ERA5-L estimated SPEI shows a weak relationship (CC = 0.35) and error (RMSE = 1.1) with RG observation. However, the ML merging algorithms improved the SPEI values with high CC (RF = 0.91, GBM = 0.85, and KNN = 0.87) and low RMSE (RF = 0.39, GBM = 0.53, and KNN = 0.48) then SRPPs at multi-scales (Fig. 8a–c) Additionally, the novel HEML algorithms enhanced the SPEI, exhibiting elevated CC (0.92) and diminished errors (RMSE = 0.37, MAE = 0.28, RAE = 0.36) (Fig. 8a–d) Past studies also revealed that the different ML merging approaches reduced the biases of precipitation estimates for extreme event monitoring. For instance, in a recent investigation, Ghosh et al.^11^ highlighted that the double machine learning model exhibits superior performance (CC = 0.89) in contrast to satellite precipitation estimates, effectively capturing drought events in Kenya. Similarly, Citakoglu and Coskun^84^ documented the outstanding performance of the ensemble ML model in capturing drought events in Turkey.

### Evaluation of super drought characteristics

#### Super drought duration

**Figure 9:**
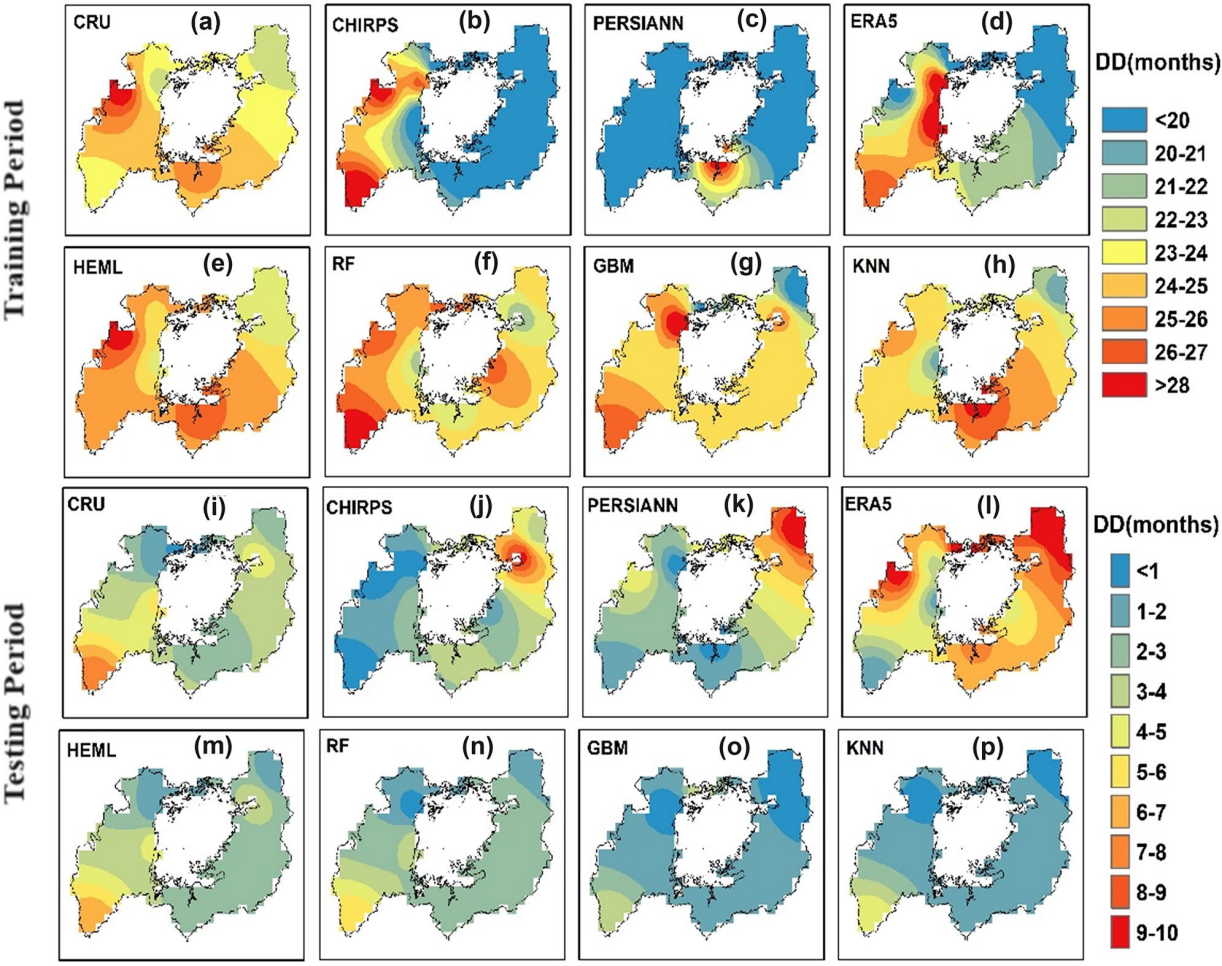
Spatial distribution of DD map showing the number of drought month during the period of 1984–2019 at SPI 3-time scale using ArcGIS 10.8.

Figure 9 represents the spatial pattern of super drought duration (DD) from SRPPs, single ML, and HEML estimated SPEI-3 in LVB during training (1984–2009) and testing period (2010–2019). A higher super DD (> 28 months) was observed during the testing period in the northwestern part of LVB. The SRPPs estimated super DD shows underestimated values in most regions, indicating poor performance. In contrast, the CHIRPS and ERA5-L results show the overestimated values in the eastern part of the studied region (Fig. 9d). In addition, the individual ML model shows an overestimated/underestimated spatial pattern of DD in most of the region. It should also be noticeable that the RF overestimates DD in the eastern part of LVB (Fig. 9f). However, the newly developed HEML shows excellent performance and a similar spatial pattern for capturing the DD in LVB. Similarly, during the testing period, the HEML results perform better than individual ML merging algorithms and SRPPs (Fig. 9m). The HEML accurately captures the spatial pattern of super DD in LVB. Conversely, CHIRPS and PERSIANN-CDR exhibit overestimation in capturing the spatial pattern of DD (Fig. 9k). The performance of ERA5-L is notably poor in the Lake Victoria Basin (LVB), rendering it unsuitable for super-drought monitoring (Fig. 9l).

#### Super drought Intensity

**Figure 10:**
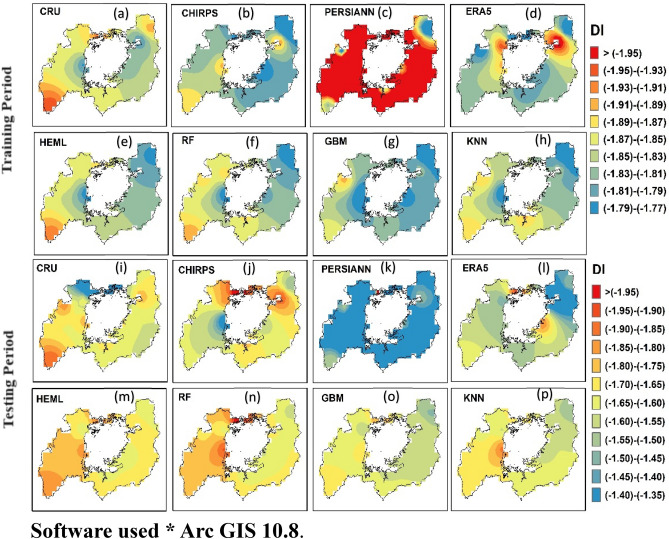
spatial distribution of drought intensity map at SPI3 time scale between 1984 and 2019 using ArcGIS 10.8.

Figure 10 illustrates the spatial pattern of super drought intensity (DI) from SRPPs, single ML, and HEML estimated SPEI-3 (short–term drought) during training (1984–2009) and testing period (2010–2019) in LVB. In the training phase, CHIRPS and ERA5-L products exhibit both overestimating and underestimating DI values in the studied region. Conversely, PERSIANN-CDR performs poorly, displaying consistent underestimation of DI (Fig. 10c). Among the ML, merging products show better results than SRPPs (Fig. 10e–m). However, the RF and HEML show excellent results for capturing the super DI in LVB (Fig. 10e–f). Similarly, PERSIANN-CDR shows overestimation and poor performance during the testing period compared to CHIRPS and ERA-L (Fig. 10k). Meanwhile, the ML (RF, KNN, GBM) merging algorithm improved the precipitation estimates for drought monitoring (Fig. 10n–p). Moreover, the HEML performs well and captures the DI during the testing period (Fig. 10m). Notably, the HEML and RF show an underestimation of DI (> − 0.3) in the northwestern part of LVB. The overall results indicate that the HEML algorithm can capture the spatial DI pattern at a regional scale.

#### Super drought frequency

**Figure 11:**
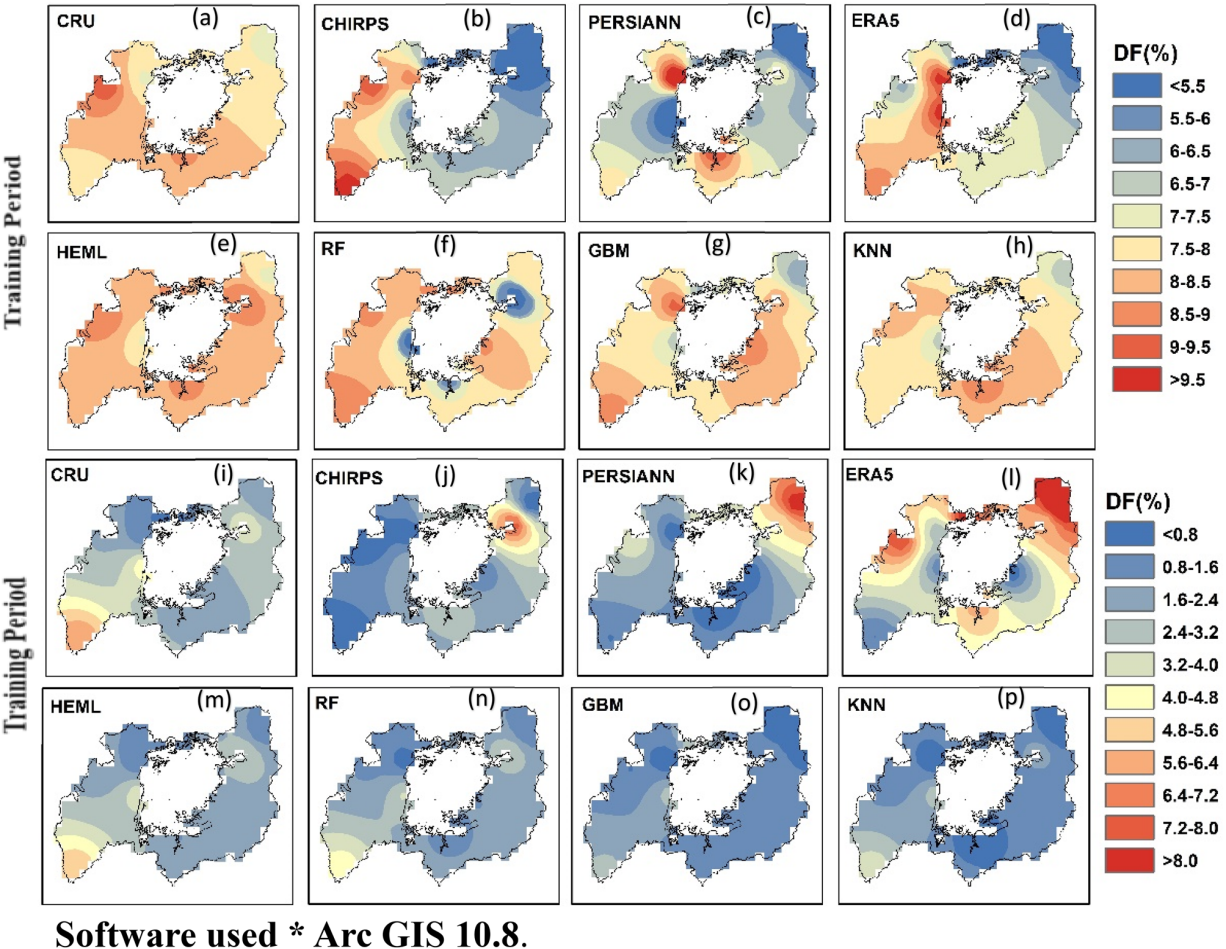
Spatial drought map showing the frequency distribution of SRPP, ML and HEML product of training and testing period at SPI3 time scale (1984–2019) using ArcGIS 10.8.

Figure 11 shows the spatial pattern of super drought frequency (DF) over LVB from SRPPs, single ML, and HEML estimated SPEI-3 (short–term drought) during training (1984–2009) and testing period (2010–2019). The SRPPs results show overestimation/underestimation for capturing the spatial pattern of Super DF in LVB. In contrast, the PERSIANN-CDR and ERA5-Land perform poorly for both the training and testing periods (Fig. 11c–d and k–l). For example, ERA5-L shows an overestimation of DF (> 8%) in most of the studied regions (Fig. 11d and l). However, the ML products perform better for capturing the DF than SRPPs in LVB (Fig. 11f–h and n–p). In addition, the HEML showed excellent performance in capturing the DF and closed with observation for the training and testing period (Fig. 11e and m). This study's results indicate that the HEML would be an alternative source of precipitation estimates in low RG density regions for accurately monitoring the drought characteristics at regional and global scales.

## Discussion

This study proposed a hybrid ML algorithm for merging global precipitation estimates and investigating the ability to capture the newly developed super drought characteristics over LVB. As far as our knowledge extends, this study represents the inaugural attempt to calculate super drought characteristics in the Lake Victoria Basin using the Hybrid Ensemble Machine-Learning (HEML) algorithm. In addition, this compares the HEML approach with traditional ML algorithms and satellite precipitation datasets. The study results clearly indicate that the HEML considerably reduces the biases of SRPPs and single ML merging products and establishes a strong relationship with observation. The findings derived from the Satellite-based Reanalysis of Precipitation Products (SRPPs) data align with previous research in the region^7,11,61^. In comparison to other machine-learning studies by Ghosh et al.^11^, Rehman et al.^31^, and Zhang et al.^37^ the outcomes of this study exhibit superior accuracy (CC = 0.93) in precipitation estimates for drought monitoring. Although the RG density influences the accuracy of precipitation estimates, the higher density provides excellent accuracy, and low density shows poor performance in that region. The poor RG density would result in a weak representation of the training period^37^. This RG density is also based on that region's climate and complex topography.

The Machine Learning (ML) algorithm consistently enhanced precipitation estimates beyond the capabilities of individual SRPPs. CHIRPS outperforms PERSIANN-CDR and ERA-5 in capturing super-drought events among the SRPPs products, as indicated by the higher CC score. The RF, KNN, and GBM merging algorithms improved the precipitation estimates and showed excellent performance for capturing the DD, DI, and DF. Moreover, the HEML algorithm shows superiority over other ML approaches and is used as an alternative source of precipitation, especially in complex topographical regions. Prior research has similarly demonstrated that employing a stacking ML algorithm enhances precipitation estimation for effective drought monitoring. For instance, in a recent investigation by Ghosh et al.^11^, it was observed that the double machine learning algorithm outperforms individual ML approaches, demonstrating superior accuracy in drought estimation in Kenya. Similarly, Prodhan et al.^4^ demonstrated the efficacy of ML algorithms in accurately estimating drought events, surpassing the performance of individual precipitation estimates. However, our findings indicate that SRPP datasets fall short of capturing the spatiotemporal patterns of drought characteristics. In contrast, the individual ML approach reported overestimation/underestimation of SPEI, which did not provide satisfactory results. The HEML algorithm adeptly replicates the spatial and temporal patterns of super-drought events in accordance with RG observations. Consequently, this study advocates for the utilisation of HEML in regional-scale drought monitoring.

The Lake Victoria Basin (LVB) is highly susceptible to drought in East Africa and has witnessed numerous drought events over recent decades^7^. The 1984 drought impacted 200,000 individuals, with a heightened frequency observed in the twenty-first century^50^. The current scenario records a staggering 40 million people affected by these extreme events, contributing to a decline in food production in the LVB^7^. Consequently, this study introduces an alternative precipitation estimation method crucial for effective drought monitoring in the area.

## Conclusion

In this study, we developed a hybrid ensemble machine learning (HEML) algorithm for merging three Satellite-based Reanalysis Precipitation Products (SRPPs) datasets—CHIRPS, PERSIANN-CDR, and ERA5-L—using three machine learning (ML) algorithms: Random Forest (RF), Gradient Boosting Machine (GBM), and K-Nearest Neighbors (KNN). This study's aim was to compare the performance of these SRPPs datasets and ML merging products to identify an alternative precipitation source for super drought computation in the Lake Victoria Basin (LVB) region. The results of our analysis demonstrate that the HEML algorithm achieves excellent accuracy (CC = 0.93) and effectively reproduces the spatial and temporal patterns of super drought characteristics, including drought intensity (DI), duration (DD), and frequency (DF) in the LVB. In contrast, the performance of the individual SRPP datasets is suboptimal, with high levels of overestimation or underestimation of Standardized Precipitation Evapotranspiration Index (SPEI) values. While the individual ML algorithms exhibit satisfactory performance, minor discrepancies in super drought characteristics were observed, though deemed negligible. The findings of our study introduce a hybrid merging method for accurate drought computation and provide a viable alternative precipitation source at the basin scale.

Furthermore, this study represents the first attempt to compute super drought characteristics (DI, DF, DD) in the LVB, contributing significantly to advancing drought research in the region. Future studies should explore the integration of additional climatic and topographical parameters, such as soil moisture, cloud properties, latitude, and longitude, into the ML merging framework. Moreover, considering all available environmental platforms for monitoring ecological changes induced by human activities is recommended to enhance drought disaster mitigation efforts. In addition, this study underscores the potential of hybrid ensemble ML algorithms for improving drought monitoring and prediction capabilities, ultimately facilitating more effective drought management strategies and enhancing resilience to extreme climate events in the Lake Victoria Basin.

### Supplementary Information

Supplementary Table S1.

## Data Availability

The study utilised publicly available datasets, which can be accessed at the following links: CHIRPS precipitation data at https://www.chc.ucsb.edu/data/chirps, climate data from the Climatic Research Unit (CRU) at https://sites.uea.ac.uk/cru/data, and ERA-Interim reanalysis datasets from the European Centre for Medium-Range Weather Forecasts (ECMWF) at https://www.ecmwf.int/en/forecasts/datasets/reanalysis-datasets/era-interim. Also, data supporting the findings of this study are derived from the corresponding author upon request.
