# Supplementary material for: A hybrid ensemble learning merging approach for enhancing the super drought computation over Lake Victoria Basin
Source: Sci Rep. 2024 Jun 15;14:13870. doi: 10.1038/s41598-024-61520-6 (PMC11180181; doi:10.1038/s41598-024-61520-6)
Supplement: Supplementary file 1 — Supplementary Table S1. [file 41598_2024_61520_MOESM1_ESM.docx]

| **Source** | **Satellite Datasets** | **Period of datasets** | **Study region** |
| --- | --- | --- | --- |
| Ayugi B et al. 2019 | PERSIAN-CDR CHIRPS, (ARC2) African Rainfall Climatology Version 2, TMPA 3B42 (Tropical Rainfall Measuring Mission Multi-Satellite Precipitation Analysis version 7) | 1998-2016 | Kenya |
| Toté C et al. (2015) | TAMSAT, ARC, TARCAT, CHIRPS | 2001-2012 | Mozambique |
| Ghosh S et al. (2024) | CHIRPS, IMERG, PERSIANN-CDR, and ERA5 | 2000–2019 | Kenya |
| Taye M et al. (2020) | (CHIRPS) and Multi-Source Weighted-Ensemble Precipitation version 2 (MSWEPv2) | 1981 to 2018 | Upper Blue Nile basin |
| Dejene NI et al. (2023) | CHIRPS, PERSIANN, PERSIANN-CCS and TMPA | 2000 to 2019 | Omo‑Gibe basin, Ethiopia |
| CATTANI E et al. (2016) | CMORPH (Climate Prediction Center MORPHING technique), JAXA (Japan Aerospace Exploration Agency), TMPA3B42, PERSIANN, TARCAT (Tropical Applications of Meteorology using satellite and ground-based observations (TAMASAT) African Rainfall Climatology and Time series), | 2001–2009 | East Africa |
| Perk S et al. (2020) | MODIS, TRMM | 2000-2016 | East Africa |
| Das P et al. (2022) | CHIRPS, PERSIANN-CDR | 1984-2020 | Lake Victoria Basin (LVB) |
| Lemma E et al. (2022) | CHIRPS | 1982-2016 | African countries, Ethiopia and major river basins of Ethiopia |

**Table S1. Detail description of** **satellite-based reanalysis precipitation products (SRPPs) used for drought monitoring in East Africa**
